# Positive regulation of the *Shewanella oneidensis* OmpS38, a major porin facilitating anaerobic respiration, by Crp and Fur

**DOI:** 10.1038/srep14263

**Published:** 2015-09-18

**Authors:** Tong Gao, Lili Ju, Jianhua Yin, Haichun Gao

**Affiliations:** 1 Institute of Microbiology and College of Life Sciences, Zhejiang University, Hangzhou, Zhejiang, 310058, China

## Abstract

Major porins are among the most abundant proteins embedded in the outer membrane (OM) of Gram-negative bacteria, playing crucial roles in maintenance of membrane structural integrity and OM permeability. Although many OM proteins (especially *c*-type cytochromes) in *Shewanella oneidensis*, a research model for respiratory versatility, have been extensively studied, physiological significance of major porins remains largely unexplored. In this study, we show that OmpS38 and OmpA are two major porins, neither of which is responsive to changes in osmolarity or contributes to the intrinsic resistance to β-lactam antibiotics. However, OmpS38 but not OmpA is largely involved in respiration of non-oxygen electron acceptors. We then provide evidence that expression of *ompS38* is transcribed from two promoters, the major of which is favored under anaerobic conditions while the other appears constitutive. The major promoter is under the direct control of Crp, the master regulator dictating respiration. As a result, the increase in the level of OmpS38 correlates with an elevated activity in Crp under anaerobic conditions. In addition, we show that the activity of the major promoter is also affected by Fur, presumably indirectly, the transcription factor for iron-dependent gene expression.

The outer membrane (OM) of Gram-negative bacteria is a highly asymmetric barrier that hinders the permeability of both hydrophilic and hydrophobic compounds^1^. To facilitate uptake of nutrients and other molecules for growth and diverse functions of the cell, the OM is embedded with a large number of channels formed by β-barrel proteins, including porins, substrate-specific transporters, and active transporters^2^. Porins are among most abundant proteins and important constituents of the OM, comprising up to 2% of the entire protein content of the cell^1^. Classic porins (~40 kDa), exemplified by *Escherichia coli* OmpC and OmpF, consist of 12–22 transmembrane segments while small porins (<25 kDa), such as *E. coli* OmpW, have 8–10^3^,^4^. It has been well established that porins are water-filled open channels allowing the passive penetration of hydrophilic molecules, which are discriminated generally by their gross physiochemical properties, such as size, hydrophobicity, and charge^5^,^6^. As bacterial antibiotic resistance is strongly correlated with permeability changes of the OM, porins have been a research focus for their role in transporting antibiotics^7^. Additionally, porins have been implicated in respiration, electron transfer, and pathogenesis by acting as receptors for phages, adhesins, and even as virulence factors^8^,^9^,^10^,^11^,^12^.

Most biological functions with which porins are associated are attributed to major porins as they are significantly more abundant than others. Given the diverse processes in which major porins are involved, their expression is tightly regulated, often at different levels by various regulators. A well-known osmotic response regulatory system is *E. coli* EnvZ-OmpR two-component system (TCS), controlling transcription of *ompF* and *ompC* as well as a diverse set of other genes^13^,^14^,^15^. Other regulators that converge to transcriptionally regulate the differential expression of major porin genes in response to environmental disturbances including Fnr (fumarate nitrate regulator), Crp (cyclic-AMP receptor protein), Lrp (amino acid metabolism regulator), Fur (iron homeostasis regulator), and CpxA-CpxR (cell-envelope stress regulator, to name a few^16^. In addition, two small regulatory RNAs, MicF and MicC, significantly affect OmpF and OmpC production at the translational level^17^.

*Shewanella*, a group of facultative anaerobic γ-proteobacteria widely distributed in nature have received intensive attentions owing to their remarkably diverse respiratory capacities and the potential for environmental remediation and microbial fuel cell, which largely rely on extracellular electron transfer^18^,^19^,^20^. Two mechanisms are adopted by *Shewanella* to deliver electrons generated intracellularly to electron acceptors (EA) in the extracellular space: direct transfer by redox *c*-type cytochromes on the OM and indirect means mediated by electron shuttles, such as riboflavin^21^,^22^,^23^. While the OM integrity could be critical to the former, the OM permeability is important to the latter. In addition, the genus is regarded as emerging human pathogens and a reservoir for antibiotics resistance given that a number of β-lactamases and Qnr-type determinants have been isolated^24^,^25^,^26^,^27^,^28^. Hence, insights into major porins are needed because of their abundance in the OM and role in transport of hydrophilic molecules, such as electron shuttles and some antibiotics.

In *S. oneidensis* MR-1, the most extensively studied *Shewanella* strain, a large number of porins have been predicted in the genome annotation^29^. No known biological function has been demonstrated for these putative porins except for SO_3896, which is found to have a role in anaerobic respiration and its promoter is one of the most robust^12^,^30^. For regulation, the bacterium contains an *E. coli* EnvZ-OmpR analogue, but it has diverged considerably from its *E. coli* counterpart despite its connection with osmoregulation^13^,^31^. In this study we first made efforts to identify and functionally characterize the major porins in *S. oneidensis*. Our results demonstrate that two porins, SO_3896 and SO_3545, named as OmpS38 and OmpA respectively, stand out as the most abundant ones. Interestingly, neither is notably responsive to changes in osmolarity or required for transport of β-lactam antibiotics into the periplasm. Further investigation reveals that OmpS38 is generally important in respiration of non-oxygen EAs and that the enhanced expression during anaerobic respiration largely relies on Crp activation.

## Materials and Methods

### Bacterial strains, plasmids, and culture conditions

A list of all bacterial strains and plasmids used in this study is given in Table 1. All chemicals were acquired from Sigma Co. (Shanghai, China) unless otherwise noted. Information for primers used in this study is available upon request. For genetic manipulation, *E. coli* and *S. oneidensis* strains under aerobic conditions were grown in Lysogeny Broth (LB, Difco, Detroit, MI) medium (tryptone, 10 g/L; yeast extract, 5 g/L; and NaCl, 5 g/L) at 37 and 30 °C, respectively. When needed, the growth medium was supplemented with chemicals at the following concentrations: 2, 6-diaminopimelic acid (DAP), 0.3 mM; ampicillin sodium, 50 μg/ml; kanamycin sulfate, 50 μg/ml; and gentamycin sulfate; 15 μg/ml.

### Physiological characterization of *S. oneidensis* strains

MS defined medium with 30 mM sodium lactate as the carbon source was used for growth and respiration characterization as described previously^32^. For anaerobic respiration, electron acceptors studied in this study included nitrate (3 mM), fumarate (20 mM), trimethylamine *N*-oxide (TMAO, 20 mM), ferric citrate (10 mM), and goethite (FeO(OH), 10 mM). Growth of *S. oneidensis* strains under aerobic or anaerobic conditions was determined by recording optical densities of cultures at 600 nm (OD_600_). For aerobic growth, fresh medium was inoculated to ~0.01 of OD_600_ with overnight cultures grown from a single colony. For anaerobic growth, cultures of mid-log phase (~0.2 of OD_600_, the same through the study) grown aerobically were centrifuged, washed with fresh medium twice, purged in nitrogen and suspended in fresh medium to ~0.01 of OD_600_ in an anaerobic glove box. To cultivate ∆*crp* under anaerobic conditions, TMAO was used as EA.

### Mutagenesis and genetic complementation

*S. oneidensis* in-frame deletion strains were constructed using the *att*-based Fusion PCR method^33^. In brief, two fragments flanking the target gene were generated by PCR with primers containing *attB* and the gene-specific sequence, and then joined by the second round PCR. The fusion fragments were introduced into pHGM01 by site-specific recombination using the BP Clonase (Invitrogen) according to the manufacturer’s instruction. The resulting mutagenesis vectors were transformed into *E. coli* WM3064 and then transferred into relevant *S. oneidensis* strains via conjugation. Integration of the mutagenesis constructs into the chromosome were selected by gentamycin resistance and confirmed by PCR. Verified transconjugants were grown in NaCl-less LB broth and plated on LB supplemented with 10% sucrose. Gentamycin-sensitive and sucrose-resistant colonies were screened by PCR for the intended deletion. The mutants were then verified by sequencing.

Mutants from previous studies had been successfully complemented by using either multiple-copy or single-copy (integrative) vectors (Table 1). In this study, the same complementation constructs were used and similar results were obtained as indicated in the relevant figure legends. For newly constructed mutants, plasmids pHG101 and pHG102 were utilized for genetic complementation^34^. For genes adjacent to their promoter, a fragment containing the coding sequence and its native promoter was generated by PCR and cloned into pHG101. For others, the coding sequence was amplified and inserted into the multiple-cloning site (MCS) of pHG102 under the control of the *arcA* promoter, which is constitutively active^35^. After verified by sequencing, the resulting complementation vector was transferred into relevant mutant strains via conjugation.

### Extraction and analysis of OM proteins

Envelope fractions were prepared according to an established method with modifications^13^,^36^. Briefly, cultures of log phase were normalized to the lowest OD to allow for comparison of OM protein quantities across strain backgrounds. Ten ml normalized culture was pelleted, washed once in 30 mM Tris-HCl (pH 8.1), and pelleted again. Cell pellets were then resuspended in 500 μl 20% sucrose in 30 mM Tris-HCl buffer (pH 8.1), followed by the addition of 20 μL of 20 mg/ml lysozyme in 0.1 mM Ethylenediaminetetraacetic acid (EDTA) (pH 7.3), and incubated on ice for 30 min. Following lysozyme treatment, 1 ml of 3 mM ETDA (pH 7.3) was added and the resulting extract was disrupted with a single 20 s pulse using a microtip sonicator (Haishu Instrument, Ningbo, China). A 1.5 ml fraction of the extract was then centrifuged at 16,000 *g* for 60 min. Envelope fractions were collected as centrifuged precipitate and resuspended in 30 μl of Laemmli SDS sample buffer. After boiled for 5 min, 15 ul samples were subjected to sodium dodecyl sulfate-polyacrylamide gel electrophoresis (SDS-PAGE, 10% acrylamide, 4 M urea, 1% SDS). Tandem mass spectrometry (MS/MS) analysis of major OM proteins was carried out essentially the same as described before^37^.

### Antibiotic and nitrite susceptibility assays

The assays were carried out as previously described^25^,^38^. In brief, cells of mid-log phase were adjusted to approximately 10^9^ cfu/ml with fresh LB and followed by 10-fold serial dilutions. Five μl of each dilution was spotted onto LB plates containing antibiotics or nitrite of varying concentrations. The plates were incubated for 24 h or longer before being read. For each strain, experiment was performed in triplicate and repeated independently at least three times.

### Chemical assays

Mid-log phase cultures were used to inoculate Bis-Tris propane (BTP) buffer (pH 5.8) with goethite as sole EA to ~0.1 of OD_600_ and incubated under anaerobic condition. Concentrations of Fe (II) of cultures were quantified using ferrozine reagent spectrophotometrically at 562 nm^39^. Standard curves were made using ferrous sulphate dissolved in 0.5 mol/L hydrochloric acid. Concentrations of nitrite in culture supernatants were measured by a modified Griess assay^40^.

### Identification of the SO_3896 transcriptional start sites

*S. oneidensis* cells were grown in LB to the mid-log phase, collected by centrifugation, and applied to RNA extraction using the RNeasy minikit (Qiagen, Shanghai) as described before^41^. RNA was quantified by using a NanoVue spectrophotometry (GE healthcare). The transcriptional start sites of SO_3896 were determined using 5′ rapid amplification of cDNA ends (RACE) according to the manufacturer’s instruction (Invitrogen, Shanghai).

### Electrophoretic motility shift assay (EMSA)

Expression and purification of *S. oneidensis* Crp protein as His-tagged fusion converted from the entire cloneset of *S. oneidensis* ORFs was described before^42^,^43^. The probes used for EMSA were prepared by PCR with ^33^P end-labeled primers. The binding reaction was performed with ~25–50 fmol (~2–5 nM) of labeled probes and various amounts of protein with or without 10 μM cAMP in 12 μl binding buffer containing 100 mM Tris/HCl (pH 7.4), 20 mM KCl, 10 mM MgCl_2_, 2 mM DTT, 0.2 μg/μL poly (dI ·dC), and 10% glycerol at 15 °C for 60 minutes and resolved on pre-run 4.8% polyacrylamide native gels^44^. The band shifts were visualized by autoradiography.

### B1H assay

Bacterial one-hydrid (B1H) system was used to investigate DNA-protein interaction *in vivo* in *E. coli* cells^45^. Briefly, plasmid constructs were created by cloning the bait DNA and target Crp into the pBXcmT and pTRG vectors, respectively, and verified by sequencing. The resultant plasmids were used to co-transform BacterioMatch II Validation Reporter Competent Cells on M9 salt agar plates containing 25 mg/ml chloramphenicol and 12.5 mg/ml tetracycline with or without 3-AT. A pair of pBXcmT-P_*cyd*_/pTRG-Crp were used as positive control, and a 300 bp DNA fragment of the 16s rRNA gene promoter (pBXcmT-P_*16s*_) was used as negative control^46^,^47^. The plates were incubated for 24 h and then moved to room temperature for an additional 16 h (colonies indicating positive interaction usually appeared between 18 and 24 h). The positive interactions were confirmed by streaking colonies on plates containing both 3-AT and streptomycin (12.5 mg/ml).

### Expression assay

Expression assay was performed using a single-copy integrative *lacZ* reporter system as described previously^48^. DNA fragments of interest were generated by PCR and cloned into the reporter vector pHGEI01, verified by sequencing, and the correct plasmid was then transferred into relevant *S. oneidensis* strains by conjugation. Once transferred into *S. oneidensis* strains, pHGEI01 containing promoter of interest integrates into the chromosome and the antibiotic marker is subsequently removed by an established approach^38^. Cells grown to the mid-log phase under experimental settings described in relevant figure legends were collected, four biological replicates in total, and levels of gene expression were determined by measuring β-Galactosidase activity essentially the same as before^38^,^48^. **Other analyses.** DNA and protein sequence similarity searches were performed using the BLAST program. Sequences of proteins of interest for alignments were obtained from Genbank. Alignments were performed using Clustal Omega (http://www.ebi.ac.uk/Tools/msa/clustalo). Signal peptide was predicted using the SignalP program^49^. The relative intensity of specific protein signals was measured using ImageJ^50^. Experimental values are subjected to statistical analyses and presented as means ± SD (standard deviation). Student’s *t*-test was performed for pairwise comparisons of groups.

### Other analyses

DNA and protein sequence similarity searches were performed using the BLAST program. Sequences of proteins of interest for alignments were obtained from Genbank. Alignments were performed using Clustal Omega (http://www.ebi.ac.uk/Tools/msa/clustalo). Signal peptide was predicted using the SignalP program^49^. The relative intensity of specific protein signals was measured using ImageJ^50^. Experimental values are subjected to statistical analyses and presented as means ± SD (standard deviation). Student’s *t*-test was performed for pairwise comparisons of groups.

## Results

### Genes SO_3545 and SO_3896 encode two major porins in *S. oneidensis*

**Figure 1:**
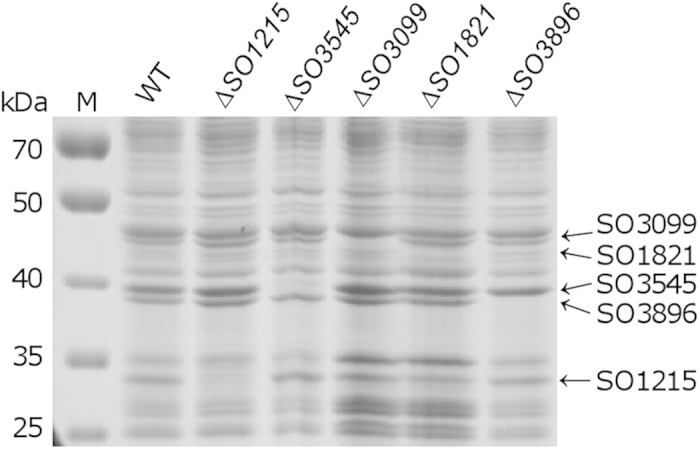
Major porins in the outer membrane (OM) in *S. oneidensis*. WT represents the wild-type strain. Cells were grown overnight in LB and subcultured 1:200 in fresh medium. OM proteins were extracted and separated on SDS-PAGE. Bands indicated are β-barrel proteins identified by MS/MS analysis and validated by comparing to the corresponding mutant. Experiments were repeated at least three times and representative results were shown.

*S. oneidensis* is predicted to be equipped with a large number of OM β-barrel proteins^29^ (Table S1). As a first step to assess biological processes in which porins are involved, we determined the profile and abundance of *S. oneidensis* OM β-barrel proteins. OM fractions were prepared from mid-log phase cells, proteins in these fractions were resolved by SDS-PAGE, and visible bands were excised for protein determination by MS/MS analysis. The identified proteins included five predicted OM β-barrel proteins, SO_1215, SO_1821, SO_3099, SO_3545, and SO_3896 (Fig. 1). The identities of these proteins were confirmed with in-frame deletion mutants for their encoding genes (Fig. 1). SO_3099 is a homologue of *E. coli* FadL, which forms a ligand-gated channel for passive diffusion of long-chain fatty acids across the OM^51^. Given that this channel mediates hydrophobic molecules, it is unlikely to be involved in transport of hydrophilic molecules as porins. Apparently, two proteins migrated close to the 40 kDa marker were most abundant. One is SO_3545 (37.4 kDa after signal peptide removal), an OmpA-like porin, which migrated at ~39 kDa (Fig. 1). The discrepancy between migration and residue-based molecular weight is common to OmpA-like porins because of posttranslational modification (PTM)^52^,^53^. Sequence analysis revealed that SO_3545 contains a segment ‘LSLGVSYRFGQGE’, which resembles the highly conserved PTM motif established in OmpA porins of other bacteria (Fig. S1). Based on these similarities, we named SO_3545 as OmpA in *S. oneidensis*. The other is SO_3896, which was recently named as OmpS38 according to its molecular weight after signal peptide removal (37.6 kDa) deduced from the sequence^30^. The last two identified OM β-barrel proteins, SO_1215 and SO_1821, are produced significantly less than OmpA or OmpS38. Currently, little is known about these two proteins, either biochemically or physiologically.

### Major porins may not be subjected to regulation by the EnvZ-OmpR system

**Figure 2:**
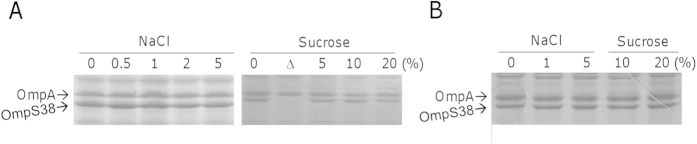
Neither OmpS38 nor OmpA responds to changes in osmolarity. (**A**) NaCl-free LB media containing NaCl or sucrose at indicated concentrations were used. The level of NaCl in the standard LB is 1%. OM proteins from cells of the similar number were extracted and separated on SDS-PAGE, Similar data were obtained from OmpA. The *ompS38* mutant (∆) was included as the control. (**B**) OM proteins extracted from the ∆*envZ*∆*ompR* strain grown under indicated conditions were applied to SDS-PAGE. Representative results were shown.

There are several mechanisms adopted by bacterial cells to cope with changes in osmolarity of the environment, one of which is through the porin proteins^1^. We thus examined production of the major porins under various osmolarities. Mid-log phase cultures were adjusted to similar cell densities, the same volume of which (containing cells of the similar number) was subjected to extraction of OM proteins. As shown in Fig. 2A, no significant difference in levels of either OmpS38 or OmpA was visualized, under conditions of both low (0 to 0.5% NaCl) and high (5% NaCl and 5 to 20% sucrose) osmolarities, suggesting that neither OmpS38 nor OmpA responds to changes in osmolarity in a significant manner. To further confirm this, we evaluated the influence of the EnvZ-OmpR system on production of OmpS38 and OmpA. As shown in Fig. 2B, levels of OmpS38 and OmpA were comparable in the ∆*envZ*∆*ompR* strain in comparison with the wild-type under all tested conditions. These data manifest that either OmpS38 or OmpA may not be a porin under regulation of the EnvZ-OmpR system.

### Major porins appear not important in diffusion of β-lactam antibiotics in *S. oneidensis*

**Figure 3:**
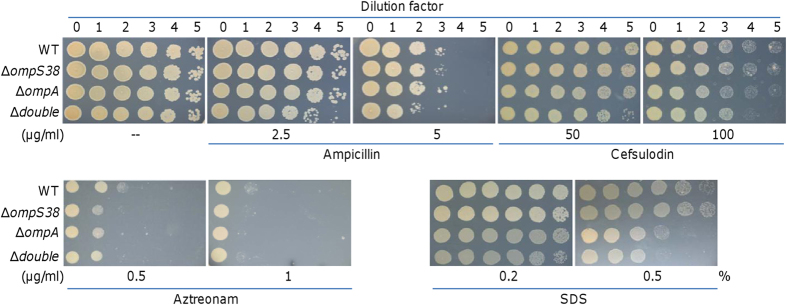
Effects of porin mutations on susceptibility to β-lactams and SDS. Strains were grown in LB to ~0.4 of OD_600_. Culture densities were normalized, 10-fold serial dilution were prepared for each, and 5 μl of each dilution was spotted onto LB without or with an chemical agent as indicated. ∆*double* represents a strain lacking both major porins. Results shown are representative of three independent experiments.

As nonspecific channels, porins form the pathway through which small, hydrophilic antibiotics, such as β-lactams, diffuse across the OM of Gram-negative bacteria^6^,^54^. As a result, loss of major classic and OmpA-like porins generally reduces the susceptibility to certain β-lactam antibiotics^55^. To test whether OmpS38 and OmpA porins play a significant role in diffusion of β-lactam antibiotics, we assayed the sensitivities of porin mutants to three different β-lactam antibiotics, ampicillin, celfsulodin, and aztreonam, in comparison with the wild-type (Fig. 3). Our previous study revealed that susceptibilities of *S. oneidensis* to these three antibiotics differ significantly^25^,^26^,^27^. Strains lacking the *ompS38* gene were indistinguishable from the wild-type in susceptibilities to all test antibiotics, indicating that OmpS38 is dispensable to diffusion of β-lactam antibiotics. Loss of OmpA resulted in a similar phenotype in general, except for slightly increased sensitivity to cefsulodin. Moreover, we constructed a strain (∆*double*) lacking both major porins and determined its susceptibility to these antibiotics. We found that the mutant displayed slightly increased sensitivity to ampicillin and cefsulodin (Fig. 3). One of possible explanations would be that loss of these two porins all together may impact the integrity of the OM, leading to faster diffusion of these antibiotics. To test this notion, we evaluated the susceptibility of these mutants to SDS, a detergent that primarily impairs the OM^36^. The results demonstrated that the lack of OmpA or both porins enhanced SDS sensitivity modestly (Fig. 3), implicating the role of OmpA in maintenance of the OM structure. In contrast, OmpS38 appeared to be negligible. Given that the loss of both major porins does not reduce susceptibilities to tested antibiotics, it is unlikely that these two major porins form the critical pathways through which β-lactams diffuse into the periplasm in *S. oneidensis*.

### OmpS38 generally affects anaerobic respiration

**Figure 4:**
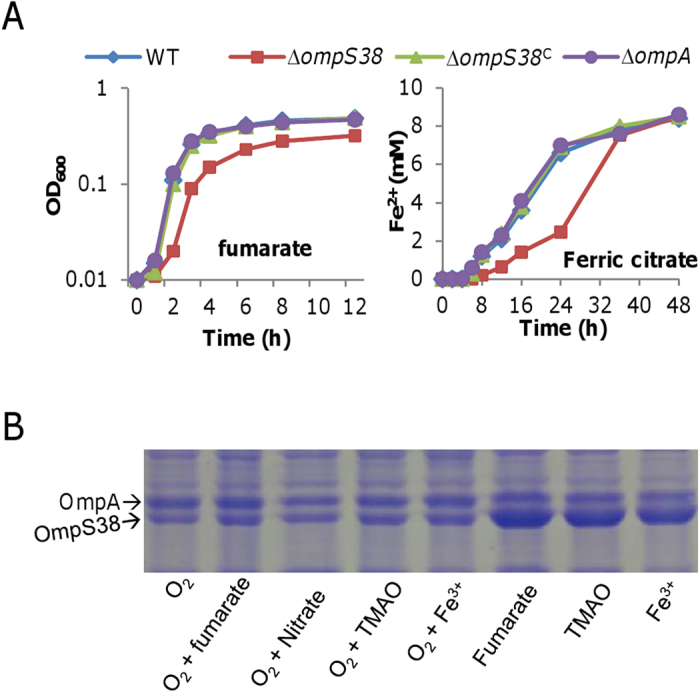
OmpS38 is involved in anaerobic respiration. (**A**) Respiration of various EAs by strains as indicated. Furamate, oxygen, and TMAO (Fig. S2) were evaluated by measuring growth whereas ferric citrate, nitrate, and FeO(OH) (Fig. S2) were estimated by measuring the levels of products as growth supported by these EAs was too low to be reliably monitored. ∆*ompS38*^C^ represents the mutant carrying a copy of the gene for complementation. Error bars (less than 10% of the average), representing S.D. from three independent experiments, were omitted for clarity. (**B**) Production of OmpS38 and OmpA under conditions indicated. Cells of mid-log phase grown on indicated EAs were subjected to SDS-PAGE. Presented are representative results of three independent experiments. Production in cultures supported by nitrate under anaerobic conditions was not assayed because growth was too poor.

OmpS38 has been previously implicated a role in respiration of Fe(III), nitrate, and fumarate^8^. To confirm this and to test whether OmpA also has a role in these processes, we compared abilities of the wild-type, *ompS38*, and *ompA* mutant strains to reduce various EAs, including oxygen, fumarate, TMAO, nitrate, ferric citrate, and insoluble FeO(OH). With all tested EAs, the ∆*ompA* strain behaved the same as the wild-type (Figs 4 and S2), eliminating the possibility that OmpA is involved in respiration of these EAs. In the case of the ∆*ompS38* strain, in comparison with the wild-type no difference was observed under aerobic condition (Fig. S2), indicating that OmpS38 has no impact on respiration of oxygen. When fumarate, TMAO, nitrate, ferric citrate, or FeO(OH) was respired, however, the ∆*ompS38* strain exhibited a consistent defect, a significant lag in respiration or growth supported by respiration (Figs 4A and S2). These data suggest that the loss of OmpS38 impedes respiration of non-oxygen EAs, at least at the early phase. Notably, the *ompS38* mutation impaired fumarate respiration also by reducing respiration rate, leading to a ~1.8–fold difference in generation time, implicating a specific mechanism other than that underlying the growth lag. Investigation into this mechanism is currently underway and will be described as part of a separate report.

To examine response of OmpS38 to these EAs, we used SDS-PAGE to assay its production. Compared to cells cultivated aerobically, those grown on fumarate, TMAO, and ferric citrate had significant enhanced production levels, which were indistinguishable from one another (Fig. 4B). Moreover, addition of these EAs to aerobic cultures did not increase OmpS38 production, indicating that oxygen, presumably simply as an EA, overwhelms the effect of these EAs. Overall, these data suggest that OmpS38 affects anaerobic respiration but may not specifically respond to non-oxygen EAs, at least in the context of expression.

### Crp is involved in regulation of *ompS38*

**Figure 5:**
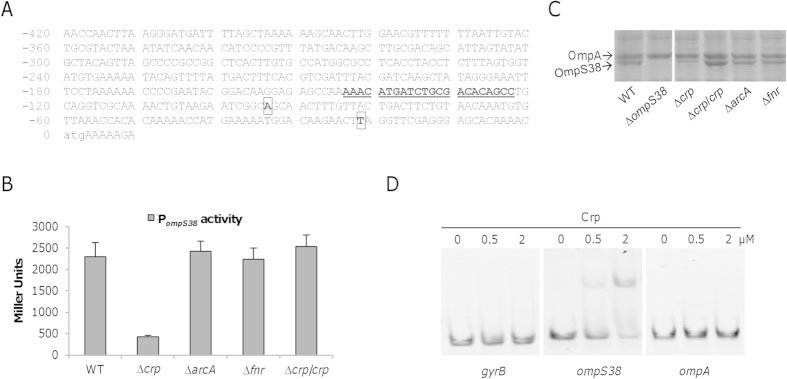
Expression of *ompS38* is under the control of Crp. (**A**) Map of the *S. oneidensis ompS38* promoter region. The Crp-binding site was in bold and underlined. The locations of transcription initiation sites are in box, which were determined by 5′-RACE described in the text (next section). (**B**) Activities of P_*ompS38*_ in strains indicated. The promoter was placed in front of *E. coli lacZ* gene and integrated into the chromosome of the strains indicated. Cells of mid-log phase were assayed by measuring β-galactosidase activity. (**C**) Production of OmpS38 in indicated strains. The lanes shown here were from the same gel, which was given in Fig. S3A. (**D**) EMSA assay was performed in the presence of 0, 0.5, or 2 μM Crp, 10 μM cAMP, and 2 nM radiolabeled promoter DNA. 0.2 μg/μl poly(dI·dC) was used in all binding reactions to block nonspecific interactions. Promoter region of *gyrB* was used as negative control. In this figure, experiments were conducted independently at least three times and error bars represent S.D. or the representative was presented.

Augmented OmpS38 production under anaerobic conditions implies an association with cellular anaerobic respiration but not with respiration of oxygen EAs. In *S. oneidensis*, Crp is the most important global regulator mediating adaptation of metabolic modes in response to the availability of electron acceptors^35^,^38^,^43^,^56^. Coincidently, a Crp-binding site was previously predicted to reside within the sequence upstream of the *ompS38* gene, from −144 to −123 relative to the translation initiation code (Fig. 5A), implying that Crp may directly regulate *ompS38* transcription^35^,^57^. To test this notion, we measured the activity of P_*ompS38*_ in the *crp*^*−*^ background using a markerless integrative *lacZ* reporter^48^. A fragment of 414 bp upstream of the coding sequence was amplified and placed in front of the full length *E. coli lacZ* gene within pHGEI01, and the resulting vector was introduced into the relevant strains. After chromosome integration and the antibiotic marker removal, activities of the promoter (P_*ompS38*_) in a single copy were assayed (Fig. 5B). The P_*ompS38*_ activity in the ∆*crp* strain was substantially reduced, approximately 20% relative to that in the wild-type, indicating that Crp is crucial for *ompS38* expression. We then tested whether the *ompS38* gene is also under control of Fnr and ArcA, two other global regulators established for mediating respiration in many bacteria^58^. However, the activity levels of P_*ompS38*_ were hardly altered in either ∆*arcA* or ∆*fnr* mutant compared with the wild-type, suggesting that neither has a significant role in regulation of the *ompS38* gene. These observations were supported by SDS-PAGE analysis of major porins (Fig. 5C). In the absence of either ArcA or Fnr, production levels of OmpS38 were statistically insignificant in comparison with the wild-type. In contrast, Crp was nearly essential for OmpS38 production as the porin was barely visible in gels stained with Coomassie Brilliant Blue. To confirm whether the Crp regulation is realized by direct interaction with the sequence upstream of the *ompS38* gene, we used EMSA and B1H assays. The reaction mixture for EMSA included DNA fragments upstream of the *ompS38* operon covering predicted Crp-binding sites, purified Crp proteins, and 10 μM cAMP, a condition proven to give the best result^43^. A similar-length fragment upstream of *gyrB* (encoding DNA gyrase subunit B) was included in the assay as negative control according to the method established previously^44^. A gel shift band was observed with the *ompS38* upstream sequences when 0.5 μM of Crp was added to the reaction mixture and the intensity of the shifted band became stronger with 2 μM of Crp (Fig. 5D). In contrast, the fragments upstream of the *gyrB* and *ompA* genes were not affected by Crp. Consistently, in the B1H assay the strong interaction was detected between Crp with the *ompS38* segment covering the predicted Crp-binding motif (Table 2). Collectively, these data converge on the idea that Crp is the regulator controlling expression of the *ompS38* gene in a direct manner.

### Transcription of *ompS38* is under control of two promoters

**Figure 6:**
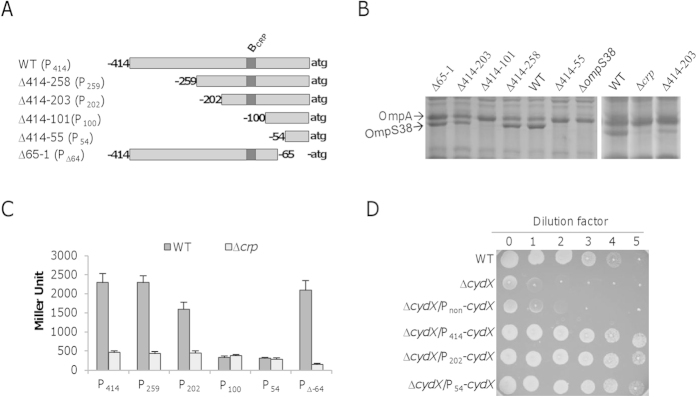
Transcription of *ompS38* initiates from two sites. (**A**) Constructs of the *S. oneidensis ompS38* promoter mutants. Constructs were named either as strains for OmpS38 production analysis shown in (**B**) or promoters for the *lacZ* reporter analysis shown in (**C**). For instance, ∆414-203 refers to a mutant lacking base-pairs from -414 to -203 relative to the *ompS38* gene and P_202_ means the resulting segment (from -202 to -1) in ∆414-203 is used to drive the *lacZ* gene. P_414_ refers to the wild-type promoter P_*ompS38*_ in Fig. 5, covering base-pairs from -414 to -1. (**B**) SDS-PAGE analysis of OM proteins in strains indicated. Gels shown here were run under the same experimental conditions. (**C**) Expression analysis of promoters indicated in WT and ∆*crp* strains. (**D**) Nitrite susceptibility assay of strains indicated. Cells were prepared as described in Fig. 3A. ∆*cydX* is hypersensitive to nitrite. P_non_, no promoter in front of *cydX*. Experiments were conducted independently at least three times and error bars represent S.D. or the representative was presented.

While loss of Crp results in substantially reduced production of OmpS38, the *ompS38* gene is still evidently expressed as revealed by both the *lacZ*-reporter and SDS-PAGE. To investigate the source for this expression, we constructed a series of mutants carrying various truncations within the region upstream of the *ompS38* coding sequence, including ∆414–258, ∆414–203, ∆414–101, and ∆414–55 (for reference, ∆414–258 represents the mutant lacking base-pairs from −414 to −259 relative to the *ompS38* gene) (Fig. 6A). The abundance of OmpS38 produced in these mutants was analyzed with SDS-PAGE (Fig. 6B). As expected, ∆414–101 and ∆414–55, in which the Crp-binding motif remains, produced OmpS38 at levels similar to that from the ∆*crp* strain. While the protein was visible in the wild-type, ∆414–258, and ∆414–203, the abundance in the former two apparently exceeded that in ∆414–203, implying that the promoter P_202_ (a fragment covering 202 bp upstream of the *ompS38* gene) is impaired with respect to activity. To confirm this, we placed these segments in front of the *E. coli lacZ* gene and measured their activities (Fig. 6A). As shown in Fig. 6C, P_414_ (from WT, 414 bp upstream of the *ompS38* gene) and P_259_ (from mutant ∆414–258) behaved identically in the the wild-type and ∆*crp* strains, indicating that these two segments are sufficiently long to support full-scale expression. Consistent with SDS-PAGE data in Fig. 6B, the promoter P_202_ had significantly reduced activity, approximately 70% relative to P_414_ and P_259_ when Crp was present. In the absence of Crp, the activity of the *ompS38* promoter within all segments tested was reduced to levels that were indistinguishable. Given that the Crp-binding motif remains, the segment probably loses another regulatory element, which is included in P_414_ and P_259_. In the case of P_100_ and P_54_, while activating effect of Crp mostly vanished, both segments drove the *lacZ* expression at levels lower than P_414_, P_259_, and P_202_ without Crp. The further reduction in activity is probably due to the complete loss of the Crp-dependent promoter. Nevertheless, the activity of approximately 300 Miller units was still obtained, which was not affected by Crp (Fig. 6C). These data together imply the presence of a weak promoter that is independent of Crp.

To provide evidence for the presence of the second *ompS38* promoter, we took advantage of our previous finding that the cytochrome *bd* oxidase confers *S. oneidensis* nitrite resistance^38^. Given that the promoter for the *bd* oxidase operon is approximately 200 Miller units, the proposed Crp-independent promoter seems sufficiently strong. We used P_54_ to drive expression of the *cydX* gene, which encodes the third functionally essential subunit of the *bd* oxidase (in addition to CydA and CydB)^59^. As shown in Fig. 6D, the *cydX* gene under the control of P_54_ restored the nitrite resistance of the ∆*cydX* strain, a scenario also obtained with P_414_ and P_202_. Given that the promoterless control vector failed to do so, it is clear that there is a promoter within 54 bp upstream of the *ompS38* gene. The presence of two promoters was then validated by 5′-RACE, as marked in Fig. 5A. The main start site (Crp-dependent) mapped to nucleotide −94 and represented 88% of the positive clones that were identified by 5′-RACE. A second site mapped to nucleotide −22. In support of these results, we constructed P_∆64_, which covers the segment from −65 to −414, and used it to drive *ompS38* expression (Fig. 6A). Based on values of protein levels and β-galactosidase activity (Fig. 6B,C), it is apparent that this promoter within the segment is strong, comparable to that within P_414_ and P_259_. Altogether, these data conclude that transcription of the *ompS38* gene initiates from two sites.

### Fur is involved in regulation of *ompS38*

**Figure 7:**
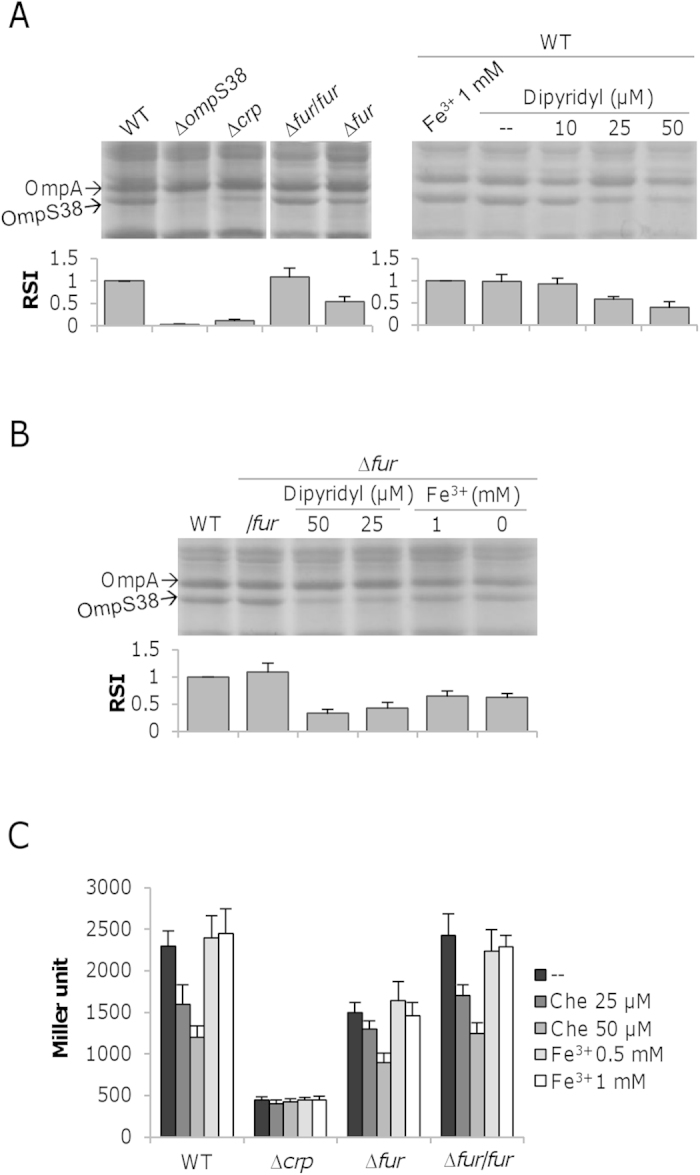
Fur is involved in regulation of *ompS38*. (**A**) SDS-PAGE analysis of OM proteins in strains indicated. The assay included the wild-type strain with iron at different levels, which were achieved by adding FeCl_3_ or iron-chelator dipyridyl. The lanes shown here were from the same gel, which was given in Fig. S3B. (**B**) SDS-PAGE analysis of OM proteins in the ∆*fur* strain under conditions indicated. In both (**A**,**B**), relative signal intensity (RSI) of OmpS38 in the indicated samples was estimated using software Image J. The averaged value for the wild-type in each gel was set to 1 as the standard, to which values of other samples were normalized. The values are the mean ± SD (error bars) (*n* = 4). (**C**) Expression analysis of P_*ompS38*_ in strains under conditions indicated. Che represents chelator dipyridyl. Experiments were conducted independently at least three times and error bars represent S.D. or the representative was presented.

The difference in *ompS38* expression levels between ∆414–203 (P_202_) and WT (P_414_) implies that the gene is subjected to additional regulation. In *Vibrio cholerae*, expression of major porin gene *ompT* is positively regulated through the direct binding by Fur, the global transcriptional regulator for iron-dependent expression of many genes^60^,^61^. Although a Fur-binding motif is not identified within the *ompS38* upstream sequence by bioinformatics prediction^57^, we made attempts to investigate whether the *ompS38* gene is transcriptionally influenced by Fur given that *V. cholerae* and *S. oneidensis* are most closely related in phylogeny, sharing extensive regions of similar gene order^29^. To this end, a ∆*fur* strain was constructed, which was validated by exhibiting a growth defect that was in excellent agreement with the results of a previous study and genetic complementation (data not shown)^62^. The influence of the Fur loss on production of OmpS38 was evident, approximately 55% relative to that in the wild-type, manifesting that the regulator functions as an activator (Fig. 7A). To examine whether the impact of Fur on expression of the *ompS38* gene is affected by intracellular iron levels, we assayed OmpS38 levels. Unlike iron in excess (Fig. 4), iron in scarcity reduced production of OmpS38, to approximately 60% with iron-chelator dipyridyl at 50 μM (Fig. 7A). However, the negative effect of dipyridyl on OmpS38 production appeared additive to that resulting from the loss of Fur (Fig. 7B). In contrast, addition of exogenous iron did not interfere with the OmpS38 production in the absence of Fur. Consistent results were obtained from the *lacZ* reporter (Fig. 7C). Expression of *ompS38* was not affected by the chelator in the ∆*crp* mutant, indicating that the loss of Crp overwhelms overall regulation. Changes in iron levels by adding dipyridyl but not exogenous iron had evident effect on the expression in the wild-type. At 50 μM, the chelator induced ∼50% reduction. A similar trend was observed in the ∆*fur* mutant. These data, collectively, suggest a possibility that the chelator and Fur function independently from each other.

## Discussion

The purpose of this study was to identify major porins and to explore how they influence the physiology in *S. oneidensis*. Our previous study had shown that the microorganism has an EnvZ-OmpR TCS, which is highly homologous to the *E. coli* counterpart^31^. Given that the system is conserved in regulation of osmolarity, we anticipated that the most abundant porins of *S. oneidensis* may share characteristics of *E. coli* OmpF and OmpC. We were surprised when this was not the case. Among five predicted β-barrel proteins revealed by combining MS/MS and mutational analyses, OmpS38 and OmpA are the most abundant but seemingly not responsive to either changes in osmolarity or the TCS. In *E. coli*, OmpA is estimated to present at ~100 000 copies per cell, making it a major OM protein crucial to cell structure and integrity^63^. Accordingly, it is not surprising that OmpA assumes the similar task in *S. oneidensis*. OmpS38 is certain to be a classic porin with 12–22 transmembrane segments as the most closely related proteins to this protein are all such porins, based on sequence similarities^8^. Additionally, the protein contains the conserved membrane core domain and variable loops typical of porins^1^. In addition to non-responsive to changes in osmolarity, the presented data showed that these two major porins are largely dispensable in allowing the entrance of β-lactam antibiotics, a function implicated in both classic and OmpA-like porins in some bacteria^7^,^55^. With respect to maintenance of the OM structure, OmpA is implicated to some extent whereas the significance of OmpS38 is negligible.

OM components are no doubt important in respiration of non-oxygen EAs, especially insoluble metal species. The OM *c*-type cytochromes complex MtrABC plays a central role in reduction of extracellular EAs acting not only as the reductase by direct contact but also as the electron station for cycling extracellular electron shuttles^23^,^64^. But how porins impact anaerobic respiration is unclear. Similar to OmpS38, a major porin OmpJ of ~49 kDa in *Geobacter sulfurreducens* was found to be essential for respiration of insoluble Fe (III) and Mn (IV) oxides^9^. Given that both OmpS38 and OmpJ lack any apparent electron transfer moieties, it is unlikely that they are involved in the final electron transfer to extracellular EAs. One possibility is that these proteins function as MtrB, which is a non-cytochrome component of the metal reduction complex. By forming a trans-OM pore of 3–4 nm in diameter^65^, MtrB allows electron transporter cytochrome *c* MtrA to span the OM such that electrons can be transferred from the periplasm to reductase MtrC, which is located on the surface of the OM (Ross *et al.* 2007). This mechanism for microbe-to-mineral electron transfer is termed as the ‘porin-cytochrome’ model^66^. In *S. oneidensis*, equally possible is that OmpS38 promotes indirect electron transfer mediated by electron shuttles such as riboflavin, another mechanism for extracellular electron transfer in this bacterium^21^,^22^. This notion gains support from a finding that *E. coli* cells producing the synthetic large pore porin protein are able to efficiently use riboflavin as the electron shuttle, resulting in significant improvement in extracellular electron transfer^11^.

The mechanism by which OmpS38 affects growth on and/or respiration of some soluble EAs, such as fumarate, nitrate, and TMAO, is not clear. Apparently, the proposal used to explain the defect in respiration of insoluble EAs does not work as these soluble EAs are reduced in the periplasm. It is possible that OmpS38 forms the major passage for extracellular small molecules across the OM to enter the periplasm. Although generally regarded to be non-specific, porins are in fact somewhat selective for small molecules, especially charged ones^5^,^67^. Thus, the OmpS38 channel may be particularly important for fumarate, leading to the substantial defect in growth on the EA in its absence. We are currently testing this possibility.

Although we have little knowledge of the mechanism by which OmpS38 is involved in anaerobic respiration, in the presented study we unraveled why expression of the porin is elevated under anaerobic conditions. In *S. oneidensis*, Crp, rather than Fnr or the Arc system, serves as the major protein mediating switch between aerobic and anaerobic respiration^35^,^56^. As activity of Crp relies on the cellular cAMP levels, which is higher under anaerobic conditions, genes under positive control of Crp are usually transcribed at augmented levels in the presence of oxygen^43^,^68^. This explains that increased expression of OmpS38 is consistently observed from cells grown on all non-oxygen EAs tested. Regulation by Crp on expression of major porins in a direct manner has been reported before in *V. cholerae* and *Yersinia pestis*^69^,^70^. However, in these enteric bacteria Crp functions as a prominent global regulator that controls the expression of an array of genes mainly in response to carbon and energy sources in the environment^71^. How these evolutionally diverse bacteria adopt the same strategy to regulate expression of major porins is of great interest.

OmpS38 is produced at reduced levels in the absence of Fur, resembling major porin OmpT of some *V. cholerae* strains^61^. Meanwhile, expression of these two porins is significantly reduced when iron is limited, suggesting a similar response to iron levels. However, differences are found. In contrast to that increased OmpT by addition of exogenous iron relies on Fur, the *S. oneidensis* regulator seems to exert positive regulation by a mechanism that is independent of iron levels. This notion is supported by a previous finding that a large number of *S. oneidensis* genes involved in energy metabolism are iron-responsive but Fur-independent^62^. Moreover, OmpS38, unlike OmpT, is probably not under direct control of Fur given the missing of a Fur-binding motif (Fur box) within its upstream region^57^,^72^.

The significance of OmpS38 expression controlled by two independent promoters is not clear. Apparently, transcription occurs from both promoters simultaneously, evidenced in the *crp* mutant strain in which the additive effect is observed. However, given that the Crp-independent promoter is substantially weaker than the Crp-dependent one, the contribution of the former to overall expression is conceivably minor. Nonetheless, because the Crp-independent promoter is seemingly constitutive, it is thus tempting to speculate that the low levels of the porin might be critical under yet unknown conditions. A goal for future experiments will be to investigate role of these promoters and to specify conditions under which their activity is required and/or altered.

## Additional Information

**How to cite this article**: Gao, T. *et al.* Positive regulation of the *Shewanella oneidensis* OmpS38, a major porin facilitating anaerobic respiration, by Crp and Fur. *Sci. Rep.* **5**, 14263; doi: 10.1038/srep14263 (2015).

## Supplementary Material

Supplementary Information

## Figures and Tables

**Table 1:** Strains and plasmids used in this study.

Strain or plasmid	Description	Reference or source
*E. coli* strains		
DH5α	Host for cloning	Lab stock
WM3064	Δ*dapA*, donor strain for conjugation	W. Metcalf, UIUC
XL1-Blue MRF’Kan	Recipient strain for one-hybrid system	Stratagene
*S. oneidensis* strains		
MR-1	Wild type	ATCC 700550
HG0624	Δ*crp* derived from MR-1	(35)
HG1215	Δ*SO1215* derived from MR-1	This study
HG1821	Δ*SO1821* derived from MR-1	This study
HG1937	*∆fur* derived from MR-1	This study
HG2356	Δ*fnr* derived from MR-1	(35)
HG3099	Δ*fadL* derived from MR-1	This study
HG3284	Δ*cydX* derived from MR-1	(59)
HG3545	Δ*ompA* derived from MR-1	This study
HG3896	Δ*ompS38* derived from MR-1	This study
HG3988	Δ*arcA* derived from MR-1	(44)
HG3545-3896	Δ*ompA*Δ*ompS38* (Δ*double*) derived from MR-1	This study
Plasmid		
pHGM01	Ap^r^, Gm^r^, Cm^r^, *att*-based suicide vector	(33)
pHG101	Km^r^, promoterless broad-host vector	(34)
pHGEI01	Km^r^, integrative *lacZ* reporter vector	(48)
pBBR-Cre	Sp^r^, helper plasmid for antibiotic cassette removal	(38)
pBXcmT	B1H bait vector	(45)
pTRG	B1H target vector	Stratagene
pHGE-P_*ompS38*_-*lacZ*	pHGEI01 containing the *ompS38* promoter	This study

**Table 2:** Bacterial one-hybrid (B1H) assay of Crp with various promoters.

Bait Vector pBXcmT	Target Vector pTRG	Colonies on nonselective plates^a^	Colonies on selective plates^b^	Confirmation^c^
/—	/—	167	0	—
/—	/Crp	176	0	—
/P_*cyd*_	/Crp	203	175	172
/P_*cyd*_	/—	163	0	—
/—	/Crp	188	0	—
/P_*16S*_	/Crp	156	1	0
/P_*ompS38*_	/Crp	144	132	128
/P_*ompA*_	/Crp	138	1	0

^a^M9 agar + 25 μg/ml chloramphenicol + 12.5 μg/ml tetracycline.

^b^a + 5 mM 3-AT.

^c^b + 12.5 μg/ml streptomycin.
