# Supplementary material for: Positive regulation of the Shewanella oneidensis OmpS38, a major porin facilitating anaerobic respiration, by Crp and Fur
Source: Sci Rep. 2015 Sep 18;5:14263. doi: 10.1038/srep14263 (PMC4585640; doi:10.1038/srep14263)
Supplement: Supplementary Information [file srep14263-s1.pdf]

Supplemental materials of

Positive regulation of the *Shewanella oneidensis* OmpS38, a major porin facilitating anaerobic respiration, by Crp and Fur

Tong Gao, Lili Ju, Jianhua Yin, and Haichun Gao*

Institute of Microbiology and College of Life Sciences, Zhejiang University, Hangzhou, Zhejiang, 310058, China

```
EcOmpA          MKKTAIAIAVALAGFATVAQAAPKDNTWYTGAKLGWSQYHD---TGFINNNGPTHENQLG 57
HiOmpA          MKKTAIALVVAGLAAASVAQAAPQENTFYAGVKAGQASFHDGLRALAREKNVGYHRNSFT 60
SO3545          MMKNTLKVVLLTSMLPLAASASQELTPWYVGAGLGVNNYEH-----IATDNG--DDNPYA 53
                * *.:: :.:     . .*.*: : ..:*.*.  *  .:..        .*   . *
EcOmpA          AGAFGGYQVN----PYVGFEMGYDWLGRMPYKG-SVENGAYKAQGVQLTAKLGYPITDDL 112
HiOmpA          YGVFGGYQILNQNNLGLAVELGYDDFGRAKGREKGKTVAKHTNHGAHLSLKGSYEVLDGL 120
SO3545          WDIFAGYMFN----DYFGAEIGYRDLGSADWTTGGISNDAG-VKGATLGLVGVWPLGNRW 108
                 . *.** .       .. *:**  :*       .        :*. *     : : :
EcOmpA          DIYTRLGGMVWRADTKSNVYG---KNHDTG-----VSPVFAGGVEYAITPEIATRLEYQW 164
HiOmpA          DVYGKAGVALVRSDYKFYEDANGTRDHKKGRHTARASGLFAVGAEYAVLPELAVRLEYQW 180
SO3545          SLSAEAGAMYYTLENSQHTGTTSSSYSSND-----FAPYVGAGVGYNFTDNLKLQAKYRR 163
                .:  . *      : .           ...      :  .. *. * .  ::  : :*:
EcOmpA          TNNIG-------DAHTIGTRPDNGMLSLGVSYRFGQGEAAPVVAPAPAPAPEVQTKHFTL 217
HiOmpA          LTRVGKYRPQDKPNTAINYNPWIGSINAGISYRFGQG-AAPVVA-----APEVVSKTFSL 234
SO3545          YENLD-------DTDFNTIEADSNYWGLELSYRFGTPAAAAPVAAAVVAAAPVDSDNDGV 216
                  .:.              ..  .  .  :*****   **. **     *. * :.   :
EcOmpA          KSD--------------------------------VLFNFNKATLKPEGQAALDQLYSQL 245
HiOmpA          NSD--------------------------------VTFAFGKANLKPQAQATLDSIYGEM 262
SO3545          YDDKDECPATPATHKVDSVGCTLYENVKKQEDVGSIQFANDSAVVKKEYYKDIERLANYM 276
                 .*                                : *  ..* :* :    :: : . :
EcOmpA          SNLDPKDGSVVVLGYTDRIGSDAYNQGLSERRAQSVVDYLISK-GIPADKISARGMGESN 304
HiOmpA          S--QVKSAKVAVAGYTDRIGSDAFNVKLSQERADSVANYFVAK-GVAADAISATGYGKAN 319
SO3545          N--KNPEFTVEIAGHASNVGKPEYNMVLSDKRADAVAKILVEKYGISQSRVTSNGYGITK 334
                .  .  . .* : *::..:*.  :*  **:.**::*.. :: * *:. . ::: * * ::
EcOmpA          PVTGNTCDNVKQRAALIDCLAPDRRVEIEVKGIKDVVTQPQA 346
HiOmpA          PVTGATCDQVKGRKALIACLAPDRRVEIAVNGTK-------- 353
SO3545          PLVAGNSKEAHAANRRIEAIVTTTEKQPVLK----------- 365
                  *:.. ...:.:     * .:..  . :  ::
```

Fig. S1. Sequence alignment of *S. oneidensis*, *E. coli*, *Haemophilus influenzae* OmpA proteins, represented as SO3545, EcOmpA, and HiOmpA, respectively. Conserved segments for posttranslational modification are in bold.

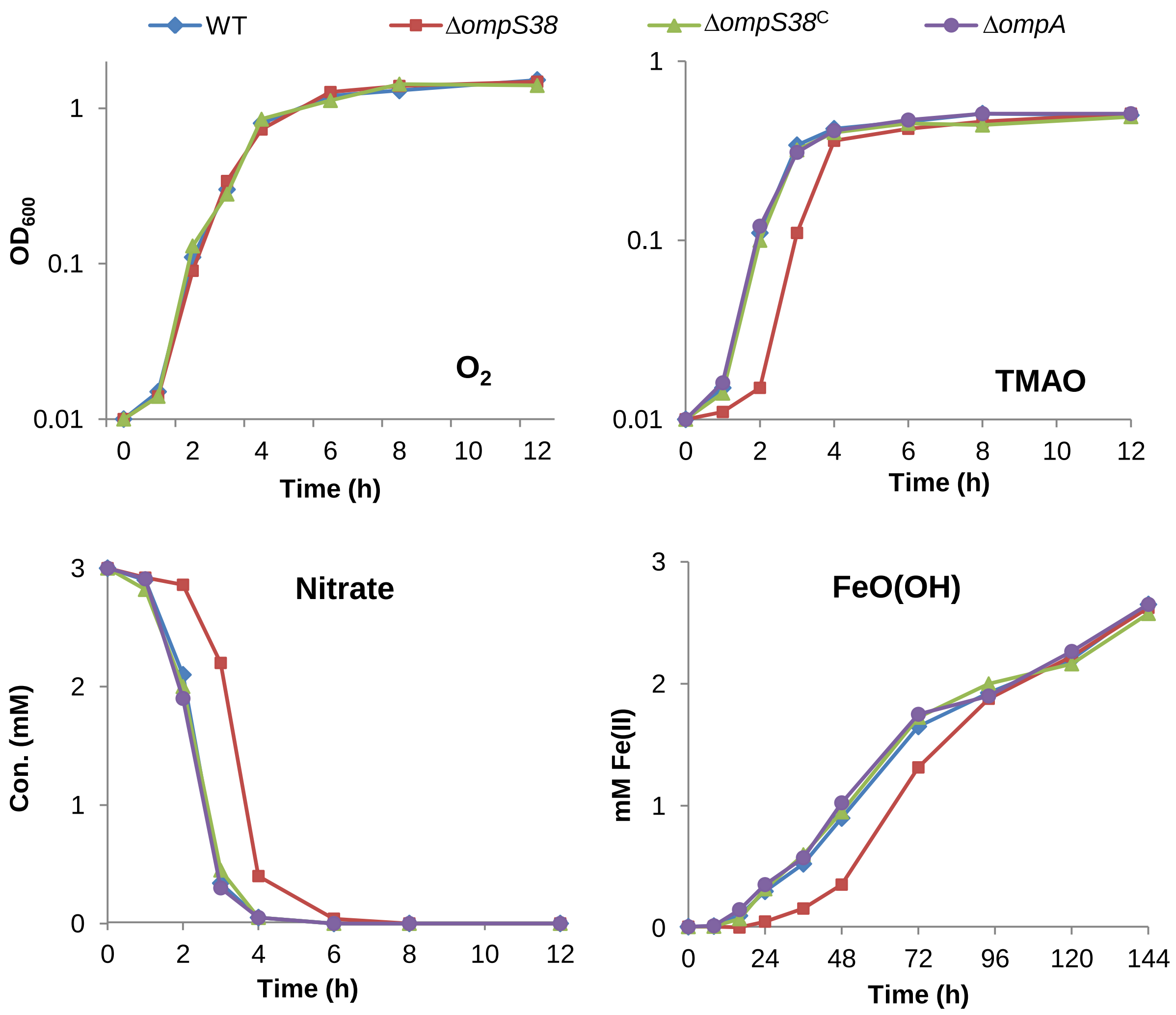

Fig. S2. OmpS38 is involved in anaerobic respiration. Respiration of various EAs by strains as indicated. Oxygen, and TMAO were evaluated by measuring growth whereas nitrate and FeO(OH) were estimated by measuring the levels of products as growth supported by these EAs was to low to be reliably monitored. Δ*ompS38*^{C} represents the mutant carrying a copy of the gene for complementation. Error bars (less than 20% of the average), representing S.D. from three independent experiments, were omitted for clarity.

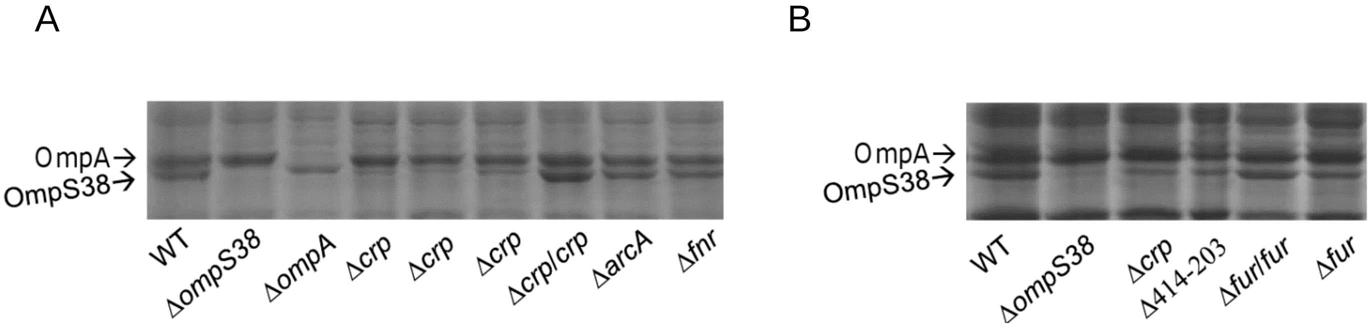

Fig. S3. Production of OmpS38 in indicated strains. (A) This is original gel for Fig. 5B. (B) This is orignal gel for Fig. 7A.

TABLE S1. Identified proteins from *S. oneidensis* outer-membrane extraction by MS/MS

No.	Pep Count	Unique PepCount	Cover Percent	MW	PI	Locus	Gene	annotation
1	361	19	72.27%	47063.42	4.53	SO_3099	fadL	Outer membrane long-chain fatty acid receptor FadL family
2	294	19	61.64%	39564.81	4.79	SO_3545		Outer membrane porin
3	187	9	44.17%	31449.82	4.71	SO_1215	ompK	Nucleoside-specific outer membrane porin OmpK
4	147	29	55.88%	48770.42	6.52	SO_1825	ttpC	TonB2 energy transduction system inner membrane component TtpC
5	109	14	70.77%	28896.79	4.94	SO_1824		TonB2 energy transduction system periplasmic component
6	52	12	38.07%	46436.4	8.77	SO_1829		Putative Zn-dependent protease associated with TonB2 energy transduction system
7	49	10	48.51%	39897.81	4.7	SO_3896	omp35	Outer membrane porin Omp35
8	38	12	37.19%	43883.47	4.5	SO_1821		Outer membrane porin
9	32	10	52.80%	31515.2	7.68	SO_4748	atpG	ATP synthase gamma chain
10	31	13	37.24%	47677.42	5.38	SO_3904	tolC	Global secretion system secretin TolC
11	8	5	26.26%	32887.54	4.84	SO_4365		Uncharacterized protein
12	8	6	23.79%	43240.01	5.72	SO_1164	dacA	D-alanyl-D-alanine carboxypeptidase DacA
13	6	2	8.78%	28414.32	6.97	SO_1105	nqrC	Na-translocating NADH-quinone reductase subunit C NqrC
14	6	2	7.69%	34802.4	6.62	SO_3802		ABC-type DrugE1 family export system ATPase component
15	5	3	23.55%	26569.38	7.85	SO_1629	rpsB	30S ribosomal protein S2
16	5	3	6.37%	58115.99	6.1	SO_3286	cydA	Cytochrome d ubiquinol oxidase subunit I CydA
17	5	3	12.69%	43284.41	5.08	SO_0217	tuf2	Elongation factor Tu 2
18	5	3	12.69%	43347.47	5.13	SO_0229	tuf1	Elongation factor Tu 1
19	5	3	11.78%	40013.05	5.68	SO_1490	adhB	Alcohol dehydrogenase II AdhB
20	5	4	20.82%	27145.14	4.72	SO_0934		Uncharacterized protein
21	4	1	3.96%	41852.41	6.42	SO_3285	cydB	Cytochrome d ubiquinol oxidase subunit II CydB
22	4	1	3.29%	42831.21	5.6	SO_1931	sucB	2-oxoglutarate dehydrogenase complex succinyl-CoA:dihydrolipoate S-succinyltransferase E2 component SucB
23	4	2	5.22%	37734.89	8.97	SO_2749	tolA	TolA energy-transducing system inner membrane component TolA
24	4	2	8.36%	38215.89	6.02	SO_1109	apbE	Fe-S assembly/repair lipoprotein ApbE
25	4	2	7.33%	46550.2	5.95	SO_3942	degQ	Periplasmic serine protease DegQ
26	4	2	6.85%	41641.24	4.98	SO_3580	bamC	Outer membrane protein assembly factor BamC
27	3	1	3.40%	52343.48	5.17	SO_4320	aggA	Type I protein secretion system secretin component AggA

No.	Pep Count	Unique PepCount	Cover Percent	MW	PI	Locus	Gene	annotation
28	3	2	15.56%	39537.43	5.32	SO_2878		Uncharacterized protein
29	3	2	8.68%	34954.92	5.26	SO_4128		Putative negative regulator of univalent cation permeability
30	3	2	4.46%	45895.27	8.8	SO_0609	petB	Cytochrome b
31	3	2	11.16%	24600.43	9.59	SO_0221	rplA	50S ribosomal protein L1
32	3	2	9.93%	31655.25	5.91	SO_1750		ABC-type transport system ATPase component
33	3	2	7.14%	40763.07	8.67	SO_1127	dnaJ	Chaperone protein DnaJ
34	3	2	7.07%	40186.07	9.07	SO_4693	vmeA	Proton-coupled multidrug efflux pump MFP component VmeA
35	3	3	11.84%	33564	9.23	SO_3748	ybiS	LD-transpeptidase YbiS
36	2	1	4.09%	40195.95	5.57	SO_3467	ribBA	Bifunctional 34-dihydroxy-2-butanone 4-phosphate synthase/GTP cyclohydrolase II RibBA
37	2	1	5.15%	31284.81	6.32	SO_4340		Putative transport protein with Tim44-like domain
38	2	1	4.40%	36114.32	6.39	SO_1743	oleB	Polyolefin biosynthetic pathway thioesterase OleB
39	2	1	2.49%	48400.29	8.89	SO_2748	tolB	Protein TolB
40	2	1	3.87%	44115.28	9.09	SO_4315	hemY	Protoheme synthesis protein HemY
41	2	1	3.27%	48080.36	5.74	SO_1926	gltA	Citrate synthase
42	2	1	2.37%	47524.68	8.3	SO_0121	ybdG	Small conductance mechanosensitive ion channel protein YbdG
43	2	1	3.86%	42074.9	5.56	SO_1897	liuA	Isovaleryl-CoA dehydrogenase LiuA
44	2	1	6.12%	30944.63	5.29	SO_1879	dapA	4-hydroxy-tetrahydrodipicolinate synthase
45	2	1	5.16%	26466.23	4.9	SO_1683	ivdG	3-hydroxyacyl-CoA dehydrogenase IvdG
46	2	1	2.75%	46799.43	5.89	SO_1948	gltP	Glutamate/aspartate:proton symporter GltP
47	2	2	8.03%	29873.24	6.02	SO_1625	dapD	2,3,4,5-tetrahydropyridine-2,6-dicarboxylate N-succinyltransferase
48	2	2	10.08%	27220.02	5.51	SO_3988	arcA	Two component signal transduction system controlling aerobic respiration response regulator ArcA
49	2	2	6.31%	40672.85	5.85	SO_1677	ivdA	3-ketoacyl-CoA thiolase IvdA
50	2	2	6.26%	49773.6	4.88	SO_4747	atpD	ATP synthase subunit beta
51	2	2	7.18%	38380.57	9.02	SO_4219	murG	UDP-N-acetylglucosamine--N-acetylmuramyl-(pentapeptide) pyrophosphoryl-undecaprenol N-acetylglucosamine transferase
52	2	2	8.16%	47317.89	8.74	SO_3517	ndh	Respiratory NADH dehydrogenase II Ndh
53	1	1	4.88%	32019.98	5.12	SO_0073		ABC-type efflux system ATPase component
54	1	1	2.38%	64228.76	5.81	SO_1928	sdhA	Succinate dehydrogenase flavoprotein subunit SdhA
55	1	1	1.92%	45282.59	6.13	SO_3471	glyA	Serine hydroxymethyltransferase

No.	Pep Count	Unique PepCount	Cover Percent	MW	PI	Locus	Gene	annotation
57	1	1	3.00%	48913.79	5.67	SO_3637	surA	Chaperone SurA
58	1	1	2.75%	46794.57	4.67	SO_4232		Outer membrane long-chain fatty acid receptor FadL family
59	1	1	3.18%	40778.25	6.52	SO_4343	agxT	Serine-pyruvate aminotransferase AgxT
60	1	1	3.62%	33812.8	5.58	SO_3185	rmlA	Glucose-1-phosphate thymidylyltransferase
61	1	1	3.31%	55148.07	5.38	SO_4749	atpA	ATP synthase subunit alpha
62	1	1	3.33%	33937.72	5.09	SO_1210	nlpI	Lipoprotein NlpI
63	1	1	3.46%	32535.33	7.67	SO_A0165		Transcriptional regulator LysR family
64	1	1	5.59%	35017.98	5.92	SO_2361	ccoP	Cbb3-type cytochrome c oxidase subunit
65	1	1	2.15%	46089.82	5.02	SO_1108	nqrF	Na(+)-translocating NADH-quinone reductase subunit F
66	1	1	4.80%	36058.57	8.01	SO_1777	mtrA	Extracelllular iron oxide respiratory system periplasmic decaheme cytochrome c component MtrA
67	1	1	5.46%	39381.19	8.58	SO_A0003		Type II restriction-modication system restriction endonuclease
68	1	1	3.51%	36997.75	6.51	SO_2779	plsX	Phosphate acyltransferase
69	1	1	5.31%	26441.32	5.56	SO_2935		Oxidoreductase short-chain dehydrogenase/reductase family
70	1	1	7.80%	22505.56	7.81	SO_4357		Extracellular oxidoreductase FeS binding subunit
71	1	1	2.31%	44686.01	5.45	SO_1214		Na+ dependent nucleoside transporter NupC family
72	1	1	2.40%	54562.84	6.23	SO_3195		Proton:peptide symporter POT family
73	1	1	6.61%	26252.58	5.53	SO_1631	pyrH	Uridylate kinase
74	1	1	4.68%	41972.71	4.98	SO_1679	ivdC	2-methylbutanoyl-CoA dehydrogenase IvdC
75	1	1	5.49%	27071.71	8.66	SO_4355		cAMP-binding regulator
76	1	1	4.55%	29057.4	8.36	SO_0904	nqrC	Na-translocating NADH-quinone reductase subunit C NqrC
77	1	1	3.62%	44112.81	5.55	SO_4118	maeB	NADP-dependent malate dehydrogenase (Oxaloacetate-decarboxylating) MaeB
